# Post-translational Modification in Control of SIRT1 Stability during DNA Damage Response

**DOI:** 10.7150/ijbs.68587

**Published:** 2022-03-27

**Authors:** Chenxi Ouyang, Guang Lu, Weifeng He, Boon-Huat Bay, Han-Ming Shen

**Affiliations:** 1 Department of Physiology, Yong Loo Lin School of Medicine, National University of Singapore, Singapore; 2 State Key Laboratory of Trauma, Burn and Combined Injury, Institute of Burn Research, Southwest Hospital, Army Medical University, Chongqing, China; 3 Department of Anatomy, Yong Loo Lin School of Medicine, National University of Singapore, Singapore; 4 Faculty of Health Sciences, University of Macau, Macau, China; 5 Ministry of Education, Frontiers Science Center for Precision Oncology, University of Macau, China

**Keywords:** DDR, SIRT1, TRIM28, caspases, ubiquitination, cleavage

## Abstract

SIRT1 (silent mating type information regulation 2 homolog 1), a class III histone deacetylase, is known to participate in multiple steps of the DNA damage response (DDR) by deacetylating several key DDR proteins. At present, the mechanisms regulating SIRT1 protein stability upon DNA damage have yet to be fully elucidated. In this study, we reveal that, under severe DNA damage, SIRT1 undergoes two forms of post-translational modifications (PTMs): (i) increased polyubiquitination and proteasomal degradation mediated by TRIM28 (tripartite motif-containing protein 28), a RING-domain E3 ligase; and (ii) cleavage at C-terminal mediated by caspases. Importantly, there is reciprocal effects between these forms of PTMs: while suppression of proteasome reduces caspases-mediated cleavage, the cleaved SIRT1 has enhanced interaction with TRIM28, thus facilitating the ubiquitination and proteasomal degradation of SIRT1. Functionally, SIRT1 works as an anti-apoptotic protein in DDR, and the above-mentioned PTMs of SIRT1 subsequently enhances cell death induced by DNA damage agents. Thus, our study has uncovered a pivotal role of SIRT1 post-translational regulation in determining cell fate in DDR.

## Introduction

DNA damage response (DDR) refers to a series of events in response to damage to the genomic DNA, including DNA damage detection, DNA damage repair pathways and cell fate decision 1. For single strand DNA damage, three repair pathways are known to be involved: mismatch repair (MMR), base excision repair (BER), and nucleotide excision repair (NER) 2. For double strand DNA damage, non-homologous end joining (NHEJ) repair and homologous recombination (HR) repair are the two principle pathways participated in the repair process. Severe DNA damage and persistent DDR signaling will lead to senescence and programmed cell death 3, 4. In mammalian cells, apoptosis is the major form of programmed cell death executed by caspases, a family of cysteine proteases 5.

Protein ubiquitination is an important form of post-translational modifications (PTMs) that is essential for various cellular processes. The ubiquitination cascade is catalyzed by ubiquitin (Ub) activating enzyme E1, Ub conjugating enzyme E2, and Ub E3 ligase 6, 7. In the group of the RING E3 ligases, tripartite motif (TRIM) proteins represent the largest sub-group 8. TRIM28, also known as KAP1 (Krüppel-Associated Box (KRAB)-Associated Protein) or TIF1-β (Transcriptional Intermediary Factor 1 β), is a multi-domain protein (110 kDa) 9. TRIM28 contributes to a variety of intracellular processes, including DDR, transcriptional co-repression, p53 degradation and autophagy 10-12. At present, there is evidence suggesting that TRIM28 is subject to different forms of PTMs in DDR. For instance, upon DNA double strand breaks (DSBs), TRIM28 is phosphorylated by ataxia telangiectasia mutated (ATM) kinase, leading to relax of the chromatin structure and promotion of DNA repair 13. Also, deacetylation of TRIM28 mediated by SIRT1 (silent mating type information regulation 2 homolog 1) stabilizes its interaction to 53BP1 and enhances NHEJ repair in cells 14.

SIRT1, a key member of the class III histone deacetylases (HADCs) family, is found predominantly in the nucleus and functions as a crucial epigenetic regulator in oxidative stress, metabolism, genome stability, senescence, autophagy, and tumorigenesis 15-17. SIRT1 causes histone deacetylation of H1K26ac, H3K9ac, H4K4ac, and H4K16ac in cells, and modulates the chromatin status and the transcriptional process 18, 19. SIRT1 also targets non-histone substrates such as p53, FOXO1 and E2F1 in response to diverse cell stress 20-22. It is known that SIRT1 plays a vital and complex role in DDR 23. For instance, SIRT1 is recruited to the damaged sites by ATM upon DSBs; in return, SIRT1 deacetylates ATM and stimulates its auto-phosphorylation and stabilization 24, 25. Meanwhile, SIRT1 targets other DDR-related proteins such as TIP60HIV-1 (Tat interactive protein 60), NBS1 (Nijmegen breakage syndrome 1), XPA and XPC (xeroderma pigmentosum group A and C), for deacetylation upon DNA damage 26-29. Thus far, the existing evidence mostly focus on the transcriptional regulation and deacetylation activity of SIRT1, while the mechanisms regulating SIRT1 protein stability by PTMs in response to DNA damage remains to be further elucidated.

In this study, we systematically examined alterations of SIRT1 protein stability upon DNA damage and the underlying mechanisms and functional implication. In response to DNA damage caused by topoisomerase inhibitors or UV exposure, SIRT1 is subject to two forms of PTMs: TRIM28-mediated ubiquitination and proteasomal degradation and caspases-mediated cleavage. Functionally, we reveal that regulation of SIRT1 protein stability plays an important role in determining the cell fate and in maintenance of genome stability in response to DNA damage.

## Materials and Methods

### Cell culture and treatments

HeLa (ATCC-CCL-2), HEK293T (ATCC-CRL-3216) and HCT116 (ATCC-CCL-247) cells were purchased from ATCC and tested to be mycoplasma-free. HeLa and HEK293T cells were cultured in Dulbecco`s modified Eagle`s medium (DMEM, HyClone, SH30022.01) supplemented with 10% fetal bovine serum (FBS, Hyclone, SV30160.03), 100 U/mL penicillin and 100 µg/mL streptomiycin (Gibco, 15140122). HCT116 cells were cultured in Roswell Park Memorial Institute (RPMI) 1640 medium (Gibco, 11875093) with 10% FBS. All cell lines were maintained in incubators with 5% CO_2_ at 37℃.

Doxorubicin (44583), Etoposide (E1383), Camptothecin (C9911), Bafilomycin A1 (B1793), Chloroquine (C6628), Cycloheximide (C7698), Q-VD-OPh (SML0063), Boc-D-FMK (B2682), KU-55933 (SML1109), EX-527/ Selisistat (E7034), and Resveratrol (R5010) were purchased from Sigma-Aldrich. MG132 (I-130-05M) was purchased from Boston Biochem. EST (330005) and ALLN (208719) were purchased from Calbiochem.

### CRISPR/Cas9 system

TRIM28 knockout HeLa cells were constructed by the CRISPR/Cas9 system. We designed three sgRNAs predicted by online prediction tool CCTop^30^: 5`- GAGCGCTTTTCGCCGCCAG -3`, 5`- CGCTGCGGGATAATGGTCGG -3`, and 5`- CTTCGAGACGCGCATGAACG -3`. Sequences were cloned into CRISPR/Cas9 vector (pSpCas9(BB)-2A-Puro (PX459)). HeLa cells were transfected with the guide RNA plasmids and cells were selected by puromycin for two weeks. Then, the cells were seeded into 24-well plates and single-cell-derived clones were picked and grown. The knockout efficiency was identified by western blot analysis and the most efficient clone was selected for further assays.

### Plasmids constructs

FLAG-SIRT1 plasmid was a kind gift from Dr. Zhu Wei-guo (Peking University Health Science Center). Subcloning was performed to generate SIRT1 constructs with different tags, including FLAG-SIRT1, EGFP-SIRT1 (pEGFP-C1 vector) and SIRT1-MYC (pcDNA4 TO myc-His A vector). Site-directed mutagenesis was generated by amplifying the plasmids with primers containing the target mutations, following the instructions in the Q5 Site-Directed Mutagenesis Kit (New England BioLabs, E0554S). All plasmid constructs were verified by DNA sequencing and immunoblotting. pSpCas9(BB)-2A-Puro (PX459), HA-TRIM28deltaRING, HA-TRIM28deltaBCC, HA-TRIM28deltaCC2, HA-TRIM28deltaLinker, HA-TRIM28deltaPB, pKH3-TRIM28, pKH3-TRIM28-C65/68A were purchased from Addgene (#62988, #124952, #124954, #124957, #124958, #124959, #45569, #92199).

### Transfection

Cells were cultured to 60%-70% confluency, then constructs were transfected using Lipofectamine 3000 (Invitrogen, L3000075) and plasmids at concentrations as per manufacturer`s instructions. After 24 h, the cells were subjected to the following treatments or harvested for distinct assays. For small interfering RNA (siRNA) transfection, cells were cultured to approximately 40%-50% confluency, then transfected with the scrambled RNAi and siRNAs targeting specific genes using Lipofectamine RNAiMAX reagent (Invitrogen, 13778150). The cells could be used for for further analyses after 48-72 h. hs.Ri.TRIM28.13.1(-2), hs.Ri.SIRT1.13.1(-3), and hs.Ri.CASP1(-10).13.1 were bought from Integrated DNA Technologies.

### Immunoblot and Immunoprecipitation

For immunoblot analysis, cells were collected and lysed in western blot lysis buffer (62.5 mM Tris-HCl pH 6.8, 25% glycerol, 2% SDS, phosphatase inhibitor and proteinase inhibitor cocktail (Thermo Fisher, 78446)). The lysates were boiled for 10 mins and cleared by centrifugation at 4℃. The samples were denatured in Laemmli sample loading buffer (Bio-Rad Laboratories, 1610737), resolved by SDS-PAGE and transferred to polyvinylidene fluoride (PVDF) membrane. After blocking with blocking buffer for 10 mins, the PVDF membrane was incubated with specific primary and secondary antibodies. Primary antibodies used in this study included those against SIRT1 (Santa Cruz Biotechnology, sc-15404, sc-74465), p-Histone H2A.X (Santa Cruz Biotechnology, sc-517348), GAPDH (Abcam, ab9485), Myc (Cell Signaling Technology, 2276), HA (Santa Cruz Biotechnology, sc-7392), GFP (Santa Cruz Biotechnology, sc-8334), PGAM5/FLAG (Invitrogen, PA5-40106), TIF1beta/KAP-1/TRIM28 (Biolegend, 619302), Phospho-TIF1-beta (Ser824) (Cell Signaling Technology, 4127), Caspase-3 (Cell Signaling Technology, 9662), Caspase 9 (Cell Signaling Technology, 9504), β-Actin (Sigma-Aldrich, A5441), Atm, phospho (Ser1981) (Santa Cruz Biotechnology, sc-47739).

The antibody-antigen complex was visualized by ECL chemiluminescence (Thermo Scientific, 34076) and quantified by the ImageQuent LAS 500 software (GE Healthcare).

For immunoprecipitation (IP), cells were lysed in IP lysis buffer (10 mM Tris-HCl pH7.4, 100 mM NaCl, 2.5 mM MgCl_2_, 0.5% Triton X-100, phosphatase inhibitor and proteinase inhibitor cocktail). The lysates were sonicated and centrifuged at 12,000 g for 10 mins. Small part of the supernatant was used as whole cell lysate sample, while 1-1.5 mg of the supernatant was transferred to a pre-chilled new tube with 10 µl anti-FLAG M2 Affinity Gel (Sigma-Aldrich, A2220), anti-c-Myc Agarose (Thermo Scientific, 20169) or GFP-Trap (chromotek, gta-20), diluted with IP lysis buffer to achieve the final concentration at 1-1.5 µg/µl, and incubated overnight at 4℃ with gentle rotation. Subsequently, the immunoprecipitates were washed 3 times by IP lysis buffer prior to elution, by boiling for 10 mins with sample loading buffer. The final eluted immunoprecipitates were subjected to SDS-PAGE and immunoblot analysis.

### Immunofluorescence staining

HeLa cells were seeded to cover glass slides with approximately 50% confluency and subjected to different treatment protocols. After fixation with 4% paraformaldehyde (PFA) and permeabilization by 0.01% Triton X-100, cells were blocked with 1% bovine serum albumin (BSA) at room temperature for more than 1 h and incubated with specific primary antibody overnight at 4℃. Proteins of interest were visualized by incubation with Alexa Fluor secondary antibody (Invitrogen, A-11029, A-11032, A-11034, A-11037) for 2 h and covered in ProLong Diamond Antifade Mountant with DAPI (Invitrogen, P36962) overnight, before observing under a Leica microscope.

### Reverse transcription and real-time quantitative PCR (qRT-PCR)

RNA was extracted from cells using RNeasy Kit according to manufacturer`s instructions (QIAGEN, 217004). Then 1 µg of the total RNA was used to perform a reverse transcription reaction with High Capacity cDNA Reverse Transcription Kit (Applied Biosystems, 4368814). Primers for the qRT-PCR are listed in Table S1. Expression of β-*ACTIN* (internal control) and* SIRT1* were examined via real-time PCR according to instructions of SsoFast EvaGreen Supermix (Bio-Rad Laboratories, 172-5201) and CFX96 Touch Real-time PCR Detection System (Bio-Rad Laboratories). For each qRT-PCR validation, three technical replicates were performed.

### *In vivo* ubiquitination assay

Cells with FLAG-SIRT1 or SIRT1-MYC overexpression were collected and lysed with denatured IP lysis buffer (50 mM Tris-HCl pH 7.4, 5 mM EDTA, 1%SDS, phosphatase inhibitor and proteinase inhibitor cocktail), boiled at 100 ℃ for 10 mins and centrifuged at 12,000 g for 10 mins. The supernatant was transferred to a new tube and 1-1.5 mg of the total protein was diluted at least 10 times volume with IP lysis buffer, so that the final concentration of SDS was less than 0.1%. Subsequently, the diluted lysates were subjected to FLAG IP or MYC IP and western blot analysis. Specific anti-K48 polyubiquitination (Cell Signaling Technology, 8081) or anti-ubiquitination (Santa Cruz Biotechnology, sc-8017) antibodies were used to determine the *in vivo* ubiquitination level.

### *In vitro* ubiquitination assay

HEK293T cells were transfected with FLAG-TRIM28 for 24-48 h, then harvested and subjected to FLAG IP. The FLAG-TRIM28 protein was trapped on FLAG beads and used for *in vitro* incubation. Recombinant His-SIRT1 was purchased from Sigma-Aldrich Technology (S8446). UBE1/UBA1 (Boston Biochem, E-305-025) and UbcH8/UBE2L6 (Boston Biochem, E2-644) were used as the E1 and E2 in the system 31. The *in vitro* 20 µl reaction mixture used was as described previously 32.

### Cell synchronization

For Nocodazole induced prometaphase arrest, HeLa cells were treated with Nocodazole (150 µg/ml, Sigma-Aldrich, M1404) for 16 h. For the double thymidine block, cells at 30-40% confluency were treated with 2.5 µM thymidine (Sigma-Aldrich, T1895) for 18 h before released into fresh medium for 8 h, followed by another 18 h treatment of 2.5 µM thymidine to synchronize the cells at G1/S boundary. Then the cells were washed three times with PBS and released to fresh medium. The cells were collected at indicated time points before western blot analysis. Cyclin B1 (Cell Signaling Technology, 12231) or AURKB/ARK2 (Santa Cruz Biotechnology, sc-25426) were used as the G2/M phase marker.

### Flow cytometry for detection of cell death

Cell death was quantified using the propidium iodide (PI)-exclusion test couple with flow cytometry. Briefly, cells were treated accordingly and collected by trypsin, then resuspended in PBS containing 50 µg/ml PI. At least 30, 000 cells were analyzed per sample by flow cytometry (BD FACSCalibur Flow Cytometer), the cell death rate was calculated by the FlowJo software.

### Nuclear and cytoplasmic Extraction

Nuclear and cytoplasmic extraction was done by NE-PER Nuclear and Cytoplasmic Extraction Reagents kit (Thermo, 78833) according to manufacturer's instructions. Lamin B (Santa Cruz Biotechnology, sc-374015) was used as the marker for the nuclear part.

### Quantification and statistical analysis

All data were quantified by the Fiji software (http://fiji.sc/) or FlowJo software (http://www.flowjo.com/solutions/flowjo) from at least three biological independent experiments and analyzed by two-tailed Student`s *t*-test for analysis of two groups and one-way ANOVA with Tukey`s multiple comparisons for analysis of three or more groups using GraphPad Prism 8 software (http://www.graphpad.com/scientific-software/prism/). Data were presented as means ± SD or means ± SEM for CHX assay. The significant levels are: *P<0.0332; **P<0.0021; ***P<0.0002; ****P<0.0001; ns, non-significant.

## Results

### SIRT1 level decreases upon DNA damage

**Figure 1:**
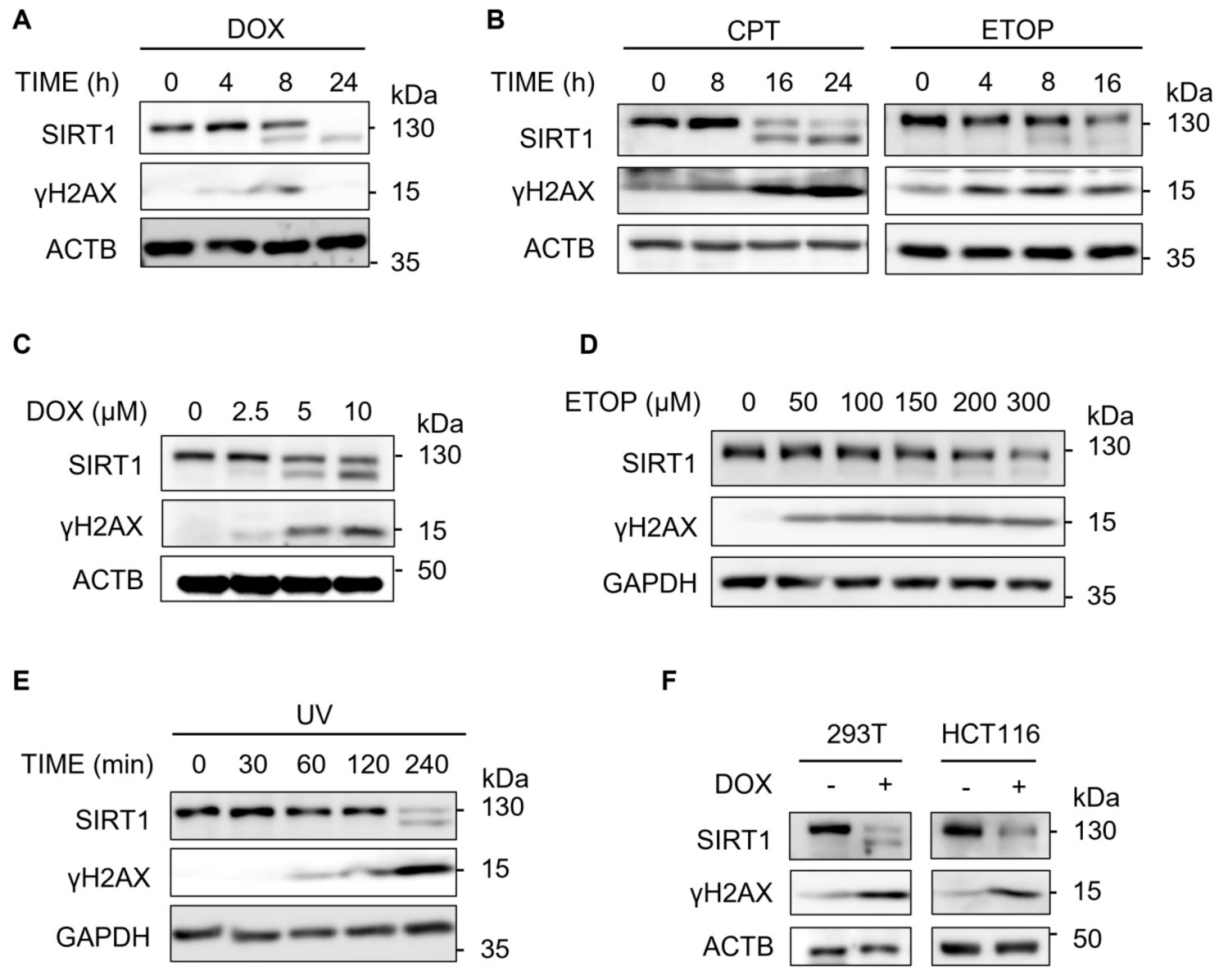
** SIRT1 level decreases upon DNA damage.** (A) HeLa cells were treated with Doxorubicin (DOX, 5µM) as indicated and cells were then harvested and cell lysate prepared and subjected to western blot analysis. (B) HeLa cells were treated with Camptothecin (CPT, 5µM) or Etoposide (ETOP, 150µM) as indicated and cells were then harvested and cell lysate prepared and subsequently subjected to western blot analysis. (C) HeLa cells were treated with DOX for indicated concentrations for 16 h before western blot analysis.(D) HeLa cells were treated with ETOP for indicated concentrations for 24 h before western blot analysis. (E) HeLa cells were exposed to UV light (UV Stratalinker 1800, Autocrosslink mode, 1200 microjoules) and incubated in fresh medium for indicated duration. Cells were then collected and analyzed by western blot analysis. (F) HEK293T cells and HCT116 cells were treated with DOX (5µM) for 24 h before western blot analysis.

In this study, we first examined the changes of the SIRT1 protein level upon DSBs induced by Doxorubicin (DOX), a topoisomerase II inhibitor 33. We observed that SIRT1 protein level decreased in a time-dependent manner in HeLa cells after treatment with DOX (Fig. 1A). γH2AX was used as a DNA damage marker 34, 35. To verify if this decrease is DOX specific, we repeated the assay using two other DNA damage agents Camptothecin (CPT) and Etoposide (ETOP) that are known to cause DSBs via their inhibitory effects on topoisomerase I and topoisomerase II, respectively 33. It turns out that both CPT and ETOP led to similar downregulation of SIRT1 in HeLa cells (Fig. 1B). In addition, DOX or ETOP caused the reduction of SIRT1 protein in a dose-dependent manner (Fig. 1C and 1D).

Previous studies have demonstrated that SIRT1 is essential in NER by targeting XPA and XPC for deacetylation 36. Consistently, we detected decreased SIRT1 level in response to UV exposure (Fig. 1E), which induces bulky DNA damage repaired mainly by NER 37. Furthermore, HEK293T cells and HCT116 cells exhibited similar pattern of downregulation of SIRT1 protein after DOX treatment (Fig. 1F), suggesting that the reduction of SIRT1 levels was not cell line specific. Taken together, downregulation of SIRT1 protein level is a common phenomenon present in multiple cell types in response to different types of DNA damage.

### SIRT1 decreases upon DNA damage in a proteasome-dependent manner

**Figure 2:**
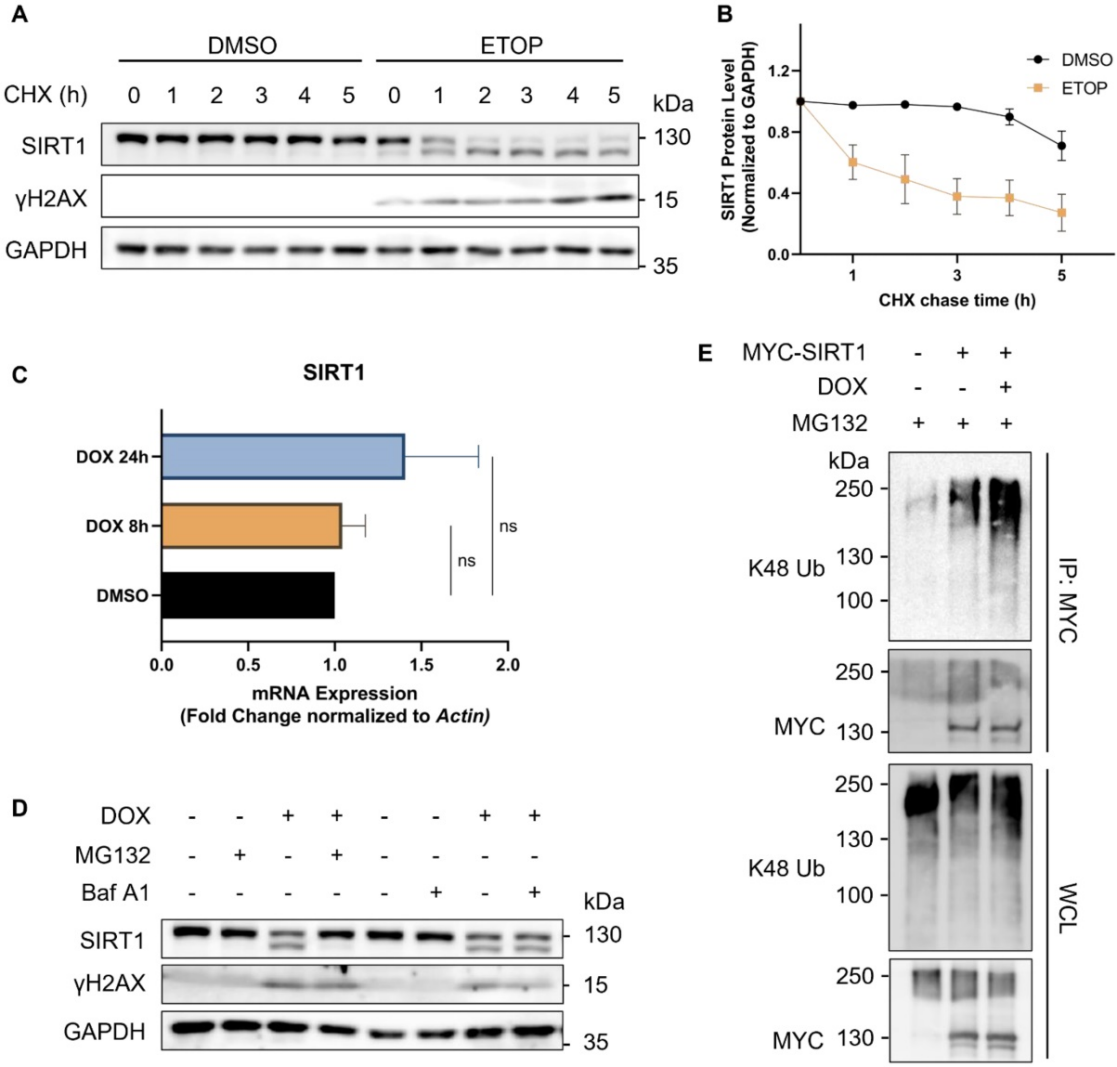
** SIRT1 protein level decreases in response to DNA damage in a proteasome-dependent manner.** (A) HeLa cells were initially treated with DMSO (the solvent control) or ETOP (150µM) for 18 h, before addition of cycloheximide (CHX, 150µg/ml), a protein synthesis inhibitor. Cells were collected every hour and subsequently subjected to western blot analysis to assess the degradation rate of SIRT1. The blot is representative of three different independent experiments. (B) Quantification of SIRT1 protein level in (A). Quantification of the western blot result was performed by the Fiji software. Data are presented as means ± SEM (n=3). (C) Total RNA was extracted from HeLa cells treated with DMSO or DOX (5µM) for 8 or 24 h. mRNA levels of *SIRT1* were quantified by qRT-PCR assay, and normalized by house-keeping gene β-Actin. (Columns, mean; error bars, ± S.D., ns, not significant; one-way ANOVA with Tukey`s multiple comparisons) (n=3). (D) HeLa cells were treated with either MG132 (20µM) or Bafilomycin A1 (Baf A1, 100µM) alone or together with DOX (5µM) as indicated for 8 h, followed by western blot analysis. (E) HEK293T cells were transfected with MYC-SIRT1 for 24 h. The cells were then treated with DMSO or DOX (5µM) together with MG132 (20µM) for 8 h and harvested for denatured IP with MYC beads. The ubiquitination level was evaluated by western blot analysis.

To determine the turnover rate of SIRT1, we blocked de novo protein synthesis using cycloheximide (CHX), a protein synthesis inhibitor. We verified that SIRT1 was more rapidly degraded in cells upon DNA damage, as evidenced by its reduced half-life following treatment of ETOP (Fig. 2A and 2B). We also checked the mRNA level of SIRT1 using real-time quantitative PCR (qRT-PCR) and did not find significant difference at the transcription level of SIRT1 in cells treated with DNA damage agents (Fig. 2C), indicating that the reduction of SIRT1 protein was due to increased protein degradation. The autophagy-lysosome system (ALS) and the ubiquitin-proteasome system (UPS) are the two major pathways involved in protein turnover 38. Thus, we applied Bafilomycin A1 (Baf A1) and MG132 to block autophagy/lysosome or proteasome-mediated degradation, respectively. Notably, only MG132, but not Baf A1, could effectively restore SIRT1 protein level in DOX-treated cells (Fig. 2D). Such results clearly suggest that the degradation of SIRT1 in DDR was UPS-dependent and independent of the ALS. Consistently, the ubiquitination level of SIRT1 was significantly elevated in DOX-treated cells (Fig. 2E), suggesting that UPS is the main mechanism underlying the downregulation of SIRT1 in DDR.

### TRIM28 interacts with SIRT1

**Figure 3:**
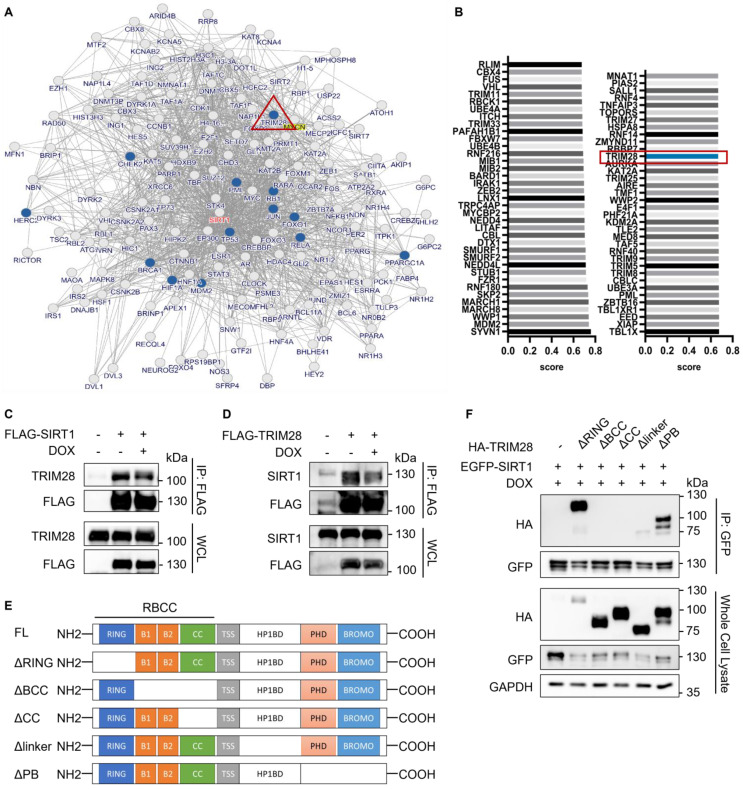
** TRIM28 is the potential E3 ligase for SIRT1 and directly interacts with SIRT1.** (A) Network view of proteins interacted with SIRT1 generated by inBio Discover, all the ubiquitination-related proteins are highlighted with blue circles. (B) Bar chart showing the potential E3 ubiquitin ligases for SIRT1 as predicted by the UbiBrower database. (C) HEK293T cells were transfected with FLAG-SIRT1 for 24 h and then treated with DMSO or DOX (5µM) for 8 h. Cell lysates were subjected to co-immunoprecipitation assay using anti-FLAG agarose and blotted with the relevant antibodies for western blot analyses. (D) HEK293T cells were transfected with FLAG- TRIM28 for 24 h and then treated with DMSO or DOX (5µM) for 8 h. Cell lysates were subjected to co-immunoprecipitation assay using anti-FLAG agarose and blotted with indicated antibodies after western blot analysis. (E) Diagrammatic representation of the truncated HA-TRIM28 plasmids structure. (F) HeLa cells were co-transfected with EGFP-SIRT1 and different HA-tagged TRIM28 truncating mutations as shown in (E) for 24 h and treated with DOX (5µM) for 8 h. Cell lysates were subjected to GFP IP and blotted with relevant antibodies.See also Figure S1.

After establishing UPS as the main mechanism responsible for reduced SIRT1 protein level in DDR, we then attempted to identify the E3 ligase targeting SIRT1 in this process. First, we used the inBio Discover protein-protein interaction database to search for ubiquitination related proteins that could interact with SIRT1 and found that TRIM28 is one such protein of interest 39 (Fig. 3A). Next, we applied the UbiBrowser software 40 to predict the potential E3 ligase for SIRT1 and the top 73 E3s are shown in Fig. 3B. TRIM28 was observed to be present in both data sets. In addition, SIRT1 was consistently present in the candidate lists from both programs when we used the two programs to predict the potential substrates of TRIM28. (Fig. S1A and S1B).

To confirm the interaction between SIRT1 and TRIM28, we performed co-immunoprecipitation assay in HEK293T cells. As predicted, TRIM28 robustly and mutually interacts with SIRT1, while DOX did not alter the interaction, indicating that the interaction between TRIM28 and SIRT1 is constitutive (Fig. 3C and 3D). Additionally, the immune-histofluorescence analysis in HeLa cells showed that both TRIM28 and SIRT1 were mainly localized in nucleus in both control and DOX-treated cells (Fig. S1C). To identify the interacting domain of TRIM28 with SIRT1, we developed five truncated HA-TRIM28 constructs with deletion of the RING, B-box, Coiled-Coil, HP1BD linker, or PHD/BROMO domain, respectively (Fig. 3E). The results showed that the B-box and Coiled-Coil domain of TRIM28 are required for the interaction with SIRT1 under both normal (Fig. S1D) and DNA damage conditions (Fig. 3F).

### TRIM28 mediates SIRT1 ubiquitination and degradation

**Figure 4:**
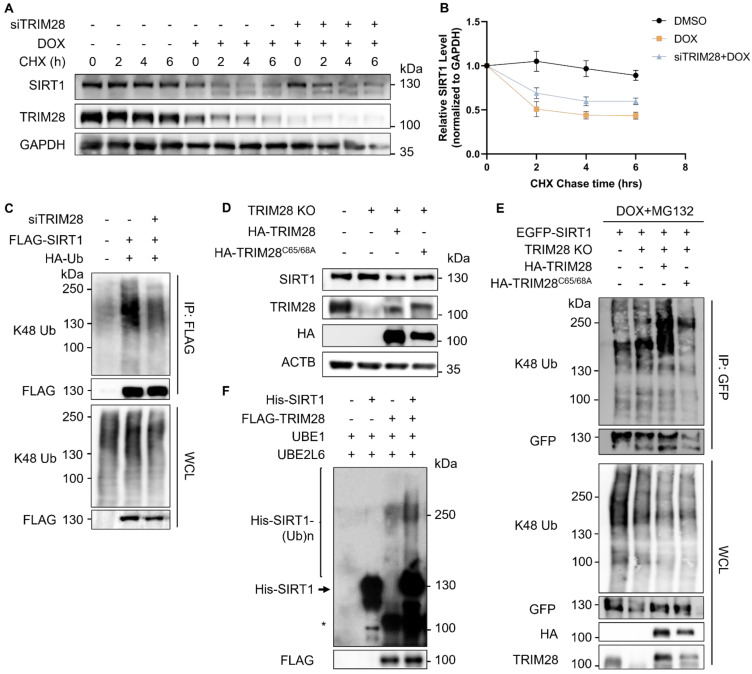
** TRIM28 mediates SIRT1 ubiquitination and proteasomal degradation.** (A) HeLa cells were transfected with siTRIM28 for two consecutive days to inhibit TRIM28 expression. CHX chase experiments were performed in control HeLa cells and TRIM28 knockdown cells treated with DMSO or DOX (5µM) for 16 hours before addition of CHX (20nM) at indicated times. SIRT1 protein level was measured by western blot analysis. The blot is representative of three different independent experiments. (B) Quantification of SIRT1 protein degradation rate in (A). The quantification was done by the Fiji software. Data are presented as means ± SEM (n=3). (C) HEK293T cells were transfected with siTRIM28 previously for one day, followed by overexpression of FLAG-SIRT1 and HA-Ub for 24 h. The cells were harvested after MG132 (10µM) treatment for 2 h and lysed with denature IP lysis buffer, before immunoprecipitation with anti-FLAG beads. (D) Wild-type HeLa cells, TRIM28 KO HeLa cells and TRIM28 KO cells transfected with HA-TRIM28 or HA-TRIM28^C65/68A^ were collected and subjected to western blot analysis with the relevant antibodies. (E) Same groups of cells in (D) were treated with DOX (5µM) and MG132 (10µM) for 8 h. The cells were lysed with denature IP lysis buffer, before immunoprecipitation with gfp-trap. (F) *In vitro* ubiquitination assay was performed using recombined His-SIRT1 protein and FLAG-TRIM28 pulled-down by FLAG-tagged agarose in HEK293T cells overexpressing FLAG-TRIM28. MYC-Ubiquitin, ATP, UBE1 and UBE2L6 (ubiquitination E2 conjugation enzyme) were used in the system and ubiquitination level was analyzed by western blot with the anti-His antibody. *, nonspecific bands. See also Figure S2.

To determine whether TRIM28 regulates the turnover of SIRT1, we performed further analysis by knocking down TRIM28. As expected, TRIM28 depletion effectively restored the SIRT1 stability in cells under DNA damage in the CHX chase experiment (Fig. 4A and 4B). Depletion of TRIM28 also led to a dramatically reduced K48-linked ubiquitination level of SIRT1 (Fig. 4C). To further validate the role of TRIM28 in control of SIRT1 protein stability, we generated TRIM28 knockout (KO) HeLa cells using the CRISPR/Cas9 system (Fig. S2A and S2B). We found increased expression of the SIRT1 protein levels in all the three TRIM28 KO clones (Fig. S2B). Moreover, TRIM28 KO effectively restored the SIRT1 protein stability in cells in the CHX chase experiment (Fig. S2C and S2D).

Next, we reconstituted both the wild-type (WT) and E3 ligase dead mutant of TRIM28 (TRIM28^C65/68A^) into the TRIM28 KO cells. As expected, reconstitution of the WT TRIM28, but not the E3 ligase activity dead mutant TRIM28^C65/68A^ markedly reduced the SIRT1 protein level (Fig. 4D). Consistently, reconstitution of the WT TRIM28 but not TRIM28^C65/68A^ showed significant enhancement of SIRT1 ubiquitination level in the *in vivo* ubiquitination assay (Fig. 4E). Finally, we confirmed the above findings by the *in vitro* ubiquitination assay using recombined His-SIRT1 protein and FLAG-TRIM28 pulled-down by anti-FLAG agarose in HEK293T cells overexpressing FLAG-TRIM28 (Fig. 4F). In summary, the above-mentioned findings demonstrate that the RING domain E3 ubiquitin ligase TRIM28 targets SIRT1 for ubiquitination and degradation.

### Caspases target SIRT1 for cleavage upon DNA damage

**Figure 5:**
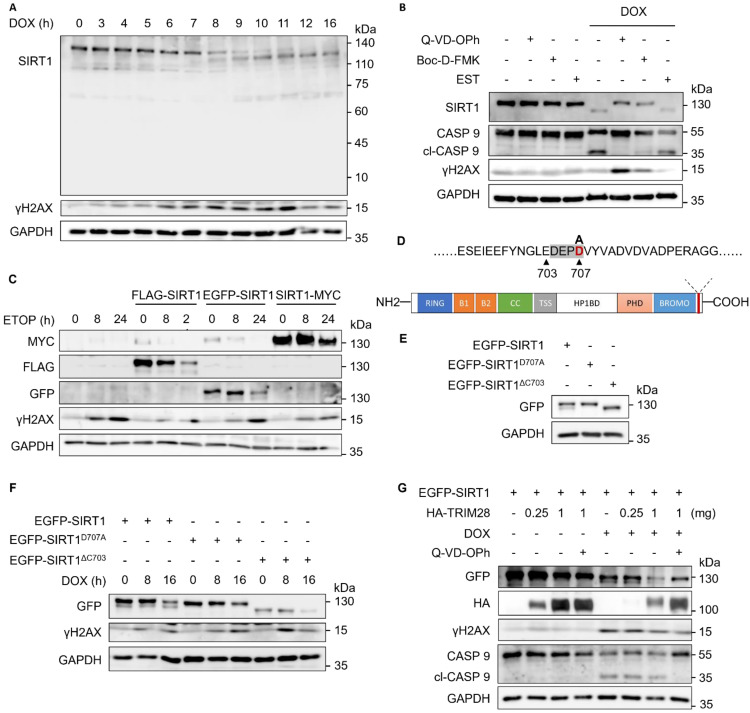
** Caspases target SIRT1 for cleavage upon DNA damage.** (A) HeLa cells were treated with DOX (5µM) for indicated hours and subjected to western blot analysis with specific antibodies.(B) HeLa cells were treated with DMSO or DOX (5µM) for 16 h with or without Q-VD-OPh (20µM), Boc-D-FMK (20µM), or EST (20µM). Cells were harvested and subjected to western blot analysis. Cleaved caspase 9 (cl-CASP 9) was used as the marker for inhibition efficiency. (C) HeLa cells were transfected with FLAG-SIRT1, EGFP-SIRT1 and SIRT1-MYC for 24 h before treated with ETOP (150µM) for indicated hours. Cell lysates were subjected to western blot analysis and detect with specific anti-FLAG/GFP/MYC antibodies. (D) Scheme of SIRT1 showing the predicted cleavage site. The potential mutation site that could abolish cleavage is labelled in red. (E) HeLa cells were transfected with EGFP-SIRT1, EGFP-SIRT1^D707A^ and EGFP-SIRT1^ΔC703^ for 24 h. Cell lysates were subjected to western blot analysis and detect with specific anti-GFP antibodies. (F) HeLa cells were transfected with EGFP-SIRT1, EGFP-SIRT1^D707A^ and EGFP-SIRT1^ΔC703^ for 24 h. The cells were treated with DOX (5µM) for the indicated hours before western blot analysis. (G) HeLa cells were transfected with EGFP-SIRT1 and HA-TRIM28 with indicated amount, and treated with DMSO, DOX (5µM) or Q-VD-Oph (20µM) for 8 h. The cells were lysed and subjected to western blot analysis. See also Figure S3.

At the beginning of the study (Fig. 1A), we noticed that apart from the degradation, a lower band of SIRT1 was observed in western blot analysis after DNA damage. We therefore confirmed the presence of this phenomenon by the time-dependent pattern in cells treated with DOX (Fig. 5A). To determine whether this down shift band was mediated by cleavage of SIRT1, we used various protease inhibitors, including pan-caspase inhibitor (z-VAD, Q-VD-OPh, Boc-D-FMK), calpain/cathepsin B inhibitor EST and calpain inhibitor ALLN. Among them, only the caspase inhibitors were able to block the formation of the lower band of SIRT1 under treatment with either DOX (Fig. 5B) or ETOP (fig. S3A), suggesting that the down shifted band was induced by caspases mediated SIRT1 cleavage. Interestingly, we observed that ALLN treatment led to SIRT1 cleavage even without DNA damage (Fig. S3A). Another interesting observation is that there is no increase of γH2AX with prolonged treatment of DOX (24 h) (Fig. 5B, also in Fig. 1A). The addition of the two caspase inhibitors prevents caspase activation and cell death and thus retained higher level of γH2AX (Fig. 5B).

Next, we knocked down caspase 1-9 (CASP 1-9) individually by siRNA transfection in HeLa cells with the knockdown efficiency shown in Fig. S3B, and then treated the cells with DOX (Fig. S3C). However, only the pan-caspase inhibitor Q-VD-OPh was able to block SIRT1 cleavage in response to DNA damage (Fig. S3C), indicating that the cleavage was mediated by multiple caspases. Moreover, we overexpressed N-terminal tagged FLAG-SIRT1 and EGFP-SIRT1, as well as C-terminal tagged SIRT1-MYC in HeLa cells. It showed that anti-FLAG and anti-GFP antibody, but not the MYC antibody, could detect two SIRT1 bands, in HeLa cells upon DNA damage (Fig. 5C), suggesting that SIRT1 is likely to be cleaved at the C-terminal.

Third, we used the online caspase site prediction tool SitePrediction to predict the possible SIRT1 cleavage site. Moreover, based on the earlier observations that the cleavage occurred at C-terminal (Fig. 5C) and the cleaved C-terminal fragment was too small to be detected in western blot (Fig. 5A), we postulated that the site DEPDVP (704-709) is the most likely cleavage site (Fig. S3D). To confirm this notion, we constructed two mutations, EGFP-SIRT1^D707A^, which could not be recognized and cleaved by caspases, and EGFP-SIRT1^ΔC703^, which was a C-terminal truncated plasmid mimicking the cleaved SIRT1 (Fig. 5D). The two mutants showed the same pattern and band sizes as the full-length SIRT1 and cleaved SIRT1 naturally occurred in cells, respectively (Fig. 5E), thus suggesting that DEPDVP (704-709) was likely the caspases-mediated cleavage site of SIRT1 in response to DNA damage. Finally, we found that both full-length and cleaved SIRT1 were degraded in response to DNA damage (Fig. 5F). Interestingly, while overexpressing TRIM28 led to a decrease of SIRT1 level with or without DOX, a pan caspase inhibitor Q-VD-OPh could partially restore the SIRT1 protein in DOX-treated cells (Fig. 5G). Such observations thus suggest a possibility that cleaved SIRT1 is more susceptible to TRIM28-mediated ubiquitination and proteasomal degradation.

### Cleavage enhances SIRT1 ubiquitination and proteasomal degradation

**Figure 6:**
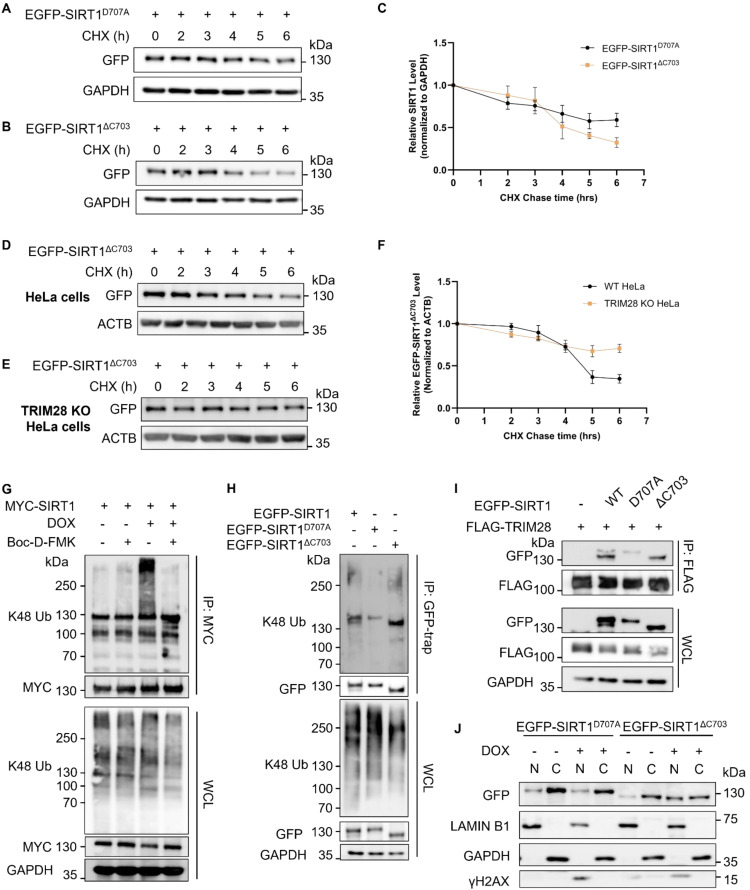
** Cleaved SIRT1 shows enhanced sensitivity to TRIM28-mediated ubiquitination and proteasomal degradation.** (A&B) HeLa cells were transfected with EGFP-SIRT1^D707A^ (A) or EGFP-SIRT1^ΔC703^ (B) for 24 h, then treated with CHX (150µg/ml). Cells were collected every hour and subsequently subjected to western blot analysis. The blot is representative of three different independent experiments. (C) Quantification of SIRT1 protein degradation rate in (A&B). Quantification of western blot result was performed by the Fiji software. Data are presented as means ± SEM (n=3). (D&E) HeLa cells (D) and TRIM28 knockout HeLa cells (E) were transfected with EGFP-SIRT1^ΔC703^ for 24 h, then treated with CHX (150µg/ml). Cells were collected every hour for western blot analysis. The blot is representative of three different independent experiments. (F) Quantification of SIRT1 protein degradation rate in (D&E). The quantification of western blot result was performed by the Fiji software. Data are presented as means ± SEM (n=3). (G) HeLa cells were transfected with MYC-SIRT1 for 24 h. Cells were treated with Boc-D-FMK (20µM) or DOX (5µM) as indicated together with MG132 (10µM) for 6 h. After which, cells were lysed with denature IP lysis buffer and subjected to immunoprecipitation with anti-MYC beads. (H) HeLa cells were transfected with EGFP-SIRT1, EGFP-SIRT1^D707A^ or EGFP-SIRT1^ΔC703^ for 24 h. Cells were treated with MG132 (10µM) for 2 h before being lysed with denature IP lysis buffer and subjected to immunoprecipitation with GFP-trap. (I) HeLa cells were co-transfected with vector, EGFP-SIRT1, EGFP-SIRT1^D707A^ or EGFP-SIRT1^ΔC703^ together with FLAG-TRIM28 for 24 h. Cell lysates were subjected to co-immunoprecipitation with anti-FLAG agarose and western blot analysis. (J) HeLa cells were transfected with EGFP-SIRT1^D707A^ or EGFP-SIRT1^ΔC703^ for 24 h, then treated with DMSO or DOX (5µM) for 6 h. Cells were harvested and subjected to cell fractionation and western blot analysis. Lamin B1 and GAPDH served as nuclear and cytoplasmic marker proteins respectively. See also Figure S4.

To examine the possibility that cleaved SIRT1 is more sensitive to TRIM28-mediated turnover, we conducted the half-life chasing assays using EGFP-SIRT1^D707A^ and EGFP-SIRT1^ΔC703^. Consistently, EGFP-SIRT1^ΔC703^ went through more rapid degradation compared to the full-length SIRT1 (Fig. 6A-6C), while the degradation speed of EGFP-SIRT1^ΔC703^ overexpressed in TRIM28 KO HeLa cells was much slower than in WT HeLa cells (Fig. 6D-6F), indicating that this rapid degradation of SIRT1 was TRIM28-dependent. In addition, Boc-D-FMK abolished the enhanced ubiquitination of SIRT1 in cells treated with DOX (Fig. 6G), further supporting the notion that cleaved SIRT1 is more susceptible to TRIM28-mediated ubiquitination. Moreover, the *in vivo* ubiquitination assays in HeLa cells showed that the SIRT1^ΔC703^ mutant that mimicked cleaved SIRT1 indeed had the highest ubiquitination level (Fig. 6H, Fig. S4A).

Next, we explored the interaction affinity between SIRT1 with different length and TRIM28. As expected, SIRT1^D707A^ showed reduced interaction with TRIM28, whereas SIRT1^ΔC703^ exhibited the opposite pattern (Fig. 6I), suggesting that the cleaved SIRT1 has higher affinity to TRIM28. Since SIRT1 is localized in both nuclei and cytoplasm, and TRIM28 is mostly in the nuclei (Fig. S1C), we next performed nuclear and cytoplasmic fraction assays in cells transfected with SIRT1^D707A^ or SIRT1^ΔC703^. SIRT1^ΔC703^ showed increased nuclear translocation in response to DNA damage (Fig. 6J). Interestingly, cleaved CASP9 could also be found in the nuclear extraction in cell under DNA damage (Fig. S4B). In summary, data from this part of our study demonstrate that SIRT1 cleavage enhances its interaction with TRIM28 and facilitates its ubiquitination and degradation.

### SIRT1 is subjected to cleavage and ubiquitination-mediated degradation in mitosis

The cell cycle (G1, S, G2 and M phase) undergoes strict regulation by respective checkpoints, while cell cycle arrest is known to play an important part in DDR 41. Here we investigated the changes of SIRT1 protein level indifferent phases of the cell cycle with two different methods to synchronize the cells. First, we used double thymidine blockage and observed that the SIRT1 protein level evidently decreased in cells when they enter mitotic stage, which was marked by enhanced Cyclin B1 and Aurora B (ARK2) (Fig. S5A). The second method was to use Nocodazole (NOC), a tubulin-binding agent that is capable of disrupting microtubule assembly and arresting cells in prometaphase of mitosis 42. Consistently, only MG132, but not ALS inhibitor chloroquine (CQ), could restore SIRT1 protein level in NOC-treated cells (Fig. S5B and S5C). Moreover, reduction of SIRT1 is not due to its transcriptional downregulation shown by the unchanged mRNA level of SIRT1 after mitotic synchronization (fig. S5D). Subsequently, the *in vivo* ubiquitination analysis revealed higher ubiquitination level of SIRT1 in mitotic cells (Fig. S5E). Moreover, the cleavage of SIRT1 also existed in mitotic cells and could be prevented by caspase inhibitor Boc-D-FMK (Fig. S5F).

Finally, TRIM28 robustly interacts with SIRT1 and treatment with NOC did not change any interaction (Fig. S5G). Consistent with this result, TRIM28 and SIRT1 were found to be colocalized in HeLa cells both in the interphase and mitotic phases, particularly, prometaphase and metaphase cells showed even higher degree of colocalization (Fig. S5H). In summary, SIRT1 in mitotic cells undergoes similar cleavage and degradation to DDR.

### SIRT1 protects cells from cell death in response to DNA damage

**Figure 7:**
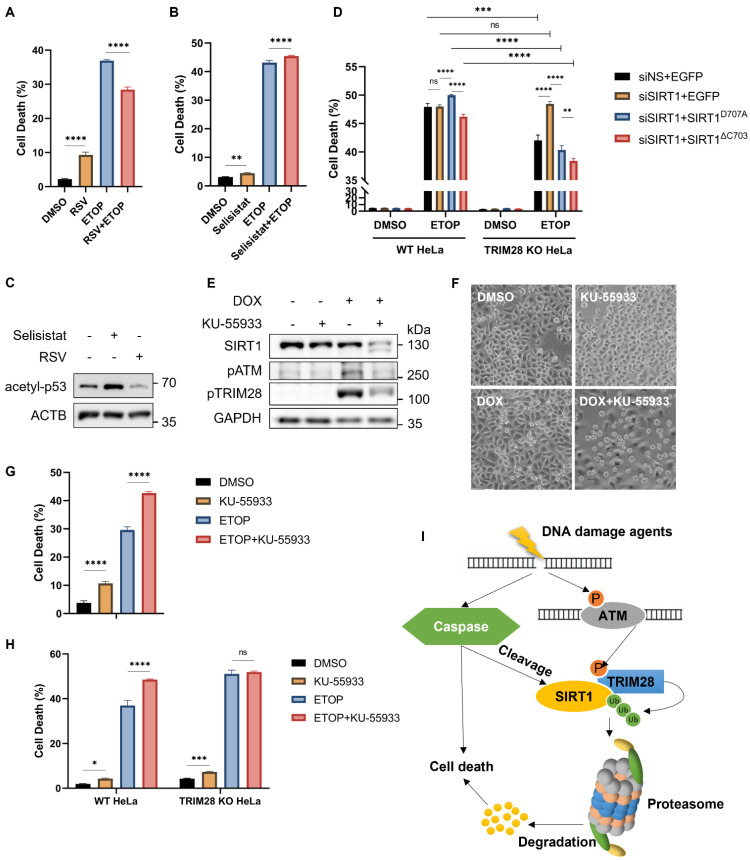
** SIRT1 protects cells from cell death in response to DNA damage.** (A) HeLa cells were treated with resveratrol (RSV, SIRT1 activator, 50µM) and ETOP (150µM) separately or collaboratively for 24 h, before cell death analysis using propidium iodide (PI) staining and flow cytometry. Data was analyzed by the FlowJo software. (B) HeLa cells were treated with Selisistat (SIRT1 inhibitor, 25µM) and ETOP (150µM) separately or collaboratively for 24 hours, before cell death analysis using PI staining and flow cytometry. Data was analyzed by FlowJo software. (C) HeLa cells were treated with RSV (50µM) and Selisistat (25µM) separately for 24 hours, then subjected to western blot analysis. Acetyl-p53 (Lys379) levels were used as indicators for SIRT1 kinase activity. (D) HeLa cells and TRIM28 KO HeLa cells were transfected with siN.S., siSIRT1, EGFP empty vector, EGFP-SIRT1^D707A^ or EGFP-SIRT1^ΔC703^ as indicated for 24 h. The cells were treated with DMSO, ETOP (250µM) for 24 h, before cell death analysis using PI staining and flow cytometry. Data was analyzed by the FlowJo software. (E&F) HeLa cells were treated with either KU-55933 (ATM inhibitor, 25µM) alone or together with DOX (5µM) for 6 h, then subjected to western blot analysis. (E). pATM (Ser1981) and pTRIM28 (Ser824) levels were used as indicators for ATM kinase activity. The morphology of the cells was observed and captured under phase-contrast microscopy (F). Scale bar: 200 µm. (G) HeLa cells were treated with either KU-55933 (25µM) alone or together with ETOP (150µM) for 24 h. before cell death analysis using PI staining and flow cytometry. Data was analyzed by the FlowJo software. (H) HeLa cells and TRIM28 KO HeLa cells were treated with KU-55933 (25µM) or ETOP (150µM) separately or collaboratively for 24 h, before cell death analysis using PI staining and flow cytometry. (I) Schematic of the novel mechanisms regulating SIRT1 stability during DNA damage response. SIRT1 is cleaved by caspases and targeted by E3 ubiquitin ligase TRIM28 for poly-ubiquitination and proteasomal degradation following severe DNA damage, rendering cell death and maintaining genome stability.(Columns, mean; error bars, ± S.D., ns, not significant; *P<0.0332, **P<0.0021, ***P<0.0002, and ****P<0.0001; one-way ANOVA with Tukey`s multiple comparisons) (n=3). See also Figure S6.

SIRT1 and TRIM28 are both multifunctional proteins involved in diverse biological processes, such as autophagy and cell death 9, 43. Here, in order to understand the functional implication of observed SIRT1 degradation in DDR, we first used Resveratrol (RSV), a known SIRT1 activator 44, to investigate the function of SIRT1 in DDR and cell fate decision. RSV-induced activation of SIRT1 attenuated the DNA damage-mediated cell death in cells treated with ETOP (Fig. 7A) or CPT (Fig. S6A). On the contrary, HeLa cells co-treated with a SIRT1 inhibitor Selisistat 45 together with DNA damage agents ETOP (Fig. 7B) or CPT (Fig. S6B) led to enhanced cell death. The efficiency of RSV and Selisistat on SIRT1 was confirmed by the altered acetyl-p53 (Lys379) level, one of the well-established substrates of SIRT1 for deacetylation 21 (Fig. 7C). Moreover, SIRT1 knockdown or overexpression (full-length or cleaved) did not affect the cell death rate upon DNA damage in the TRIM28-WT cells (Fig. 7D). However, in the TRIM28-KO HeLa cells, SIRT1 depletion significantly elevated cell death induced by ETOP, whereby this increase was abolished by overexpression of the SIRT1 (Fig. 7D). Our data thus suggest that TRIM28-mediated SIRT1 ubiquitination and degradation renders the cells more susceptible to cell death in response to DNA damage, ie, SIRT offers a protective effect against cell death induced by DNA damage agents.

It has been reported that both SIRT1 and TRIM28 are regulated by ATM in DDR 3. ATM inhibition by KU-55933, a chemical inhibitor, led to a significant decrease of SIRT1 protein level even with a shorter duration of DOX treatment compared to HeLa cells treated with DOX alone (Fig. 7E). The effectiveness of this inhibitor was shown by reduced levels of phosphorylated ATM (Ser1981) and phosphorylated TRIM28 (Ser824) (Fig. 7E). Consistently, ATM inhibition caused more cell death upon DNA damage compared to DOX treatment alone as examined by morphological changes (Fig. 7F) and by propidium iodide (PI)-exclusion test coupled with flow cytometry (Fig. 7G), suggesting that ATM plays an important pro-survival role in DDR. Such a notion was also supported by cell death data from ATM inhibitor KU-55933 in HeLa cells treated with CPT (Fig. S6C, S6D).

To determine whether TRIM28 is involved in the cell death response, we utilized TRIM28 KO HeLa cells as described earlier. In comparison to the WT cells, ATM inhibition by KU-55933 reduced SIRT1 degradation in TRIM28 KO cells (Fig. S6E and S6F), suggesting that ATM inhibition increases SIRT1 degradation upon DNA damage in a TRIM28-dependent manner. Moreover, KU-55933 enhanced cell death in ETOP-treated WT HeLa cells but not in TRIM28 KO HeLa cells judging by cell morphology and viability (Fig. S6G). Similar trends were also observed when cell death was quantified using PI-exclusion test in cells treated with ETOP (Fig. 7H) or with CPT (Fig. S6H). Taken together, the findings imply that the signaling axis of ATM-TRIM28-SIRT1 plays a key role in determining the cell fate in response to DNA damage.

## Discussion

In this study, we systematically examined the PTMs of SIRT1 in response to DNA damage and have summarized in Fig. 7I. We first established that SIRT1 is targeted by E3 ubiquitin ligase TRIM28 for ubiquitination and proteasomal degradation, in response to DNA damage caused by topoisomerase inhibitors or UV exposure. This finding is indeed consistent with earlier studies in which SIRT1 is subject to ubiquitination identified by proteomic approaches 46, 47. Two E3 ubiquitin ligases that target SIRT1 have also been identified and reported. Yu et al showed that SIRT1 is ubiquitinated by SMAD ubiquitin regulatory factor 2 (SMURF2) for degradation, leading to suppression of cell proliferation and tumorigenesis in colorectal cancer 48. In addition, MDM2 targets SIRT1 for ubiquitination both *in vivo* and *in vitro* 49. Nevertheless, neither of these studies was performed under DNA damage conditions.

Another important finding from our study is that SIRT1 could be targeted by caspases for cleavage. Interestingly, caspase-mediated SIRT1 cleavage is reminiscent of the similar cleavage of other DDR-related proteins such as DNA-PKcs (DNA-dependent protein kinase catalytic subunit) and PARP (Poly (ADP-ribose) polymerase)^50^, ^51^. Meanwhile, ATM is subjected to caspase-mediated cleavage and inactivation in apoptosis upon DNA damage 52, 53. Thus, it appears that DNA repair factors and DDR signaling proteins activated by DNA damage are likely to serve as substrates for caspase-mediated cleavage, possibly as a mechanism to promote apoptotic cell death.

After establishment of the two important forms of PTMs of SIRT1 in response to DNA damage, one interesting question remaining to be further studied is whether and how these two forms of PTMs are functionally interlinked. On one hand, suppression of proteasome function by MG132 is able to reduce caspase-mediated cleavage (Figure 2D), indicating that TRIM28-mediated SIRT1 ubiquitination may facilitate its cleavage by caspase. On the other hand, caspases-mediated SIRT1 cleavage enhances its interaction with TRIM28 and facilitates its ubiquitination and degradation (Figure 6). Therefore, it appears that these two forms of PTMs can affect each other in a reciprocal way. The exact nature of the functional interactions between TRIM28-mediated ubiquitination and caspase-mediated SIRT1 cleavage remains to be further investigated.

In this study, we identified ATM as the kinase mediating TRIM28 phosphorylation and activation, thus establishing the ATM-TRIM28-SIRT1 signaling axis in DDR, as illustrated in Figure 7I. It is known that these three proteins are functionally interlinked in response to DNA damage 13, 14, 24, 25. Thus, our findings shed new light into the intricate relationship among these three players in response to DNA damage. In our study, ATM inhibition by KU-55933 leads to enhanced SIRT1 cleavage and degradation upon DNA damage, which also results in increased cell death in HeLa cells (Fig. 7D-7F). In addition, this enhancement is largely related to the functions of TRIM28, since KU-55933 could hardly enhance SIRT1 degradation and cell death in TRIM28 knockout HeLa cells following DNA damage (Fig. 7G and Fig. S6E-S6H).

One interesting finding from this study is that SIRT1 cleavage and ubiquitination-mediated degradation also occur during mitosis. It is worth noting that TRIM28 is targeted by ATM for phosphorylation on Ser824 under both DNA damage conditions and mitosis, and this phosphorylation will disassociate TRIM28 from the heterochromatin and contribute to chromatin relaxation 9, 12. Consistently, we observed that ATM inhibition by KU-55399 results in an obvious decrease of TRIM28 Ser824 phosphorylation and increase of SIRT1 degradation (Fig. 7E and Fig. S5E-F), indicating that these two forms of PTMs of TRIM28 are functionally interlinked and the phosphorylation of TRIM28 may inhibit its E3 ligase activity.

Overall, SIRT1 is under sophisticated post-transcriptional regulations in response to DNA damage via including defined signaling axis involving TRIM28 and ATM (Fig. 7I). As an essential protein participating in DDR, understanding the regulatory mechanism of SIRT1 protein stability is crucial for ascertaining the functional interplay between DNA damage repair, cell cycle arrest, and cell death processes. Such knowledge will pave the way for the development of new interventional strategies targeting DDR signaling pathways.

## Supplementary Material

Supplementary figures and tables.
