# Supplementary material for: Post-translational Modification in Control of SIRT1 Stability during DNA Damage Response
Source: Int J Biol Sci. 2022 Mar 27;18(7):2655–69. doi: 10.7150/ijbs.68587 (PMC9066097; doi:10.7150/ijbs.68587)
Supplement: Supplementary file 1 — Supplementary figures and tables. [file ijbsv18p2655s1.pdf]

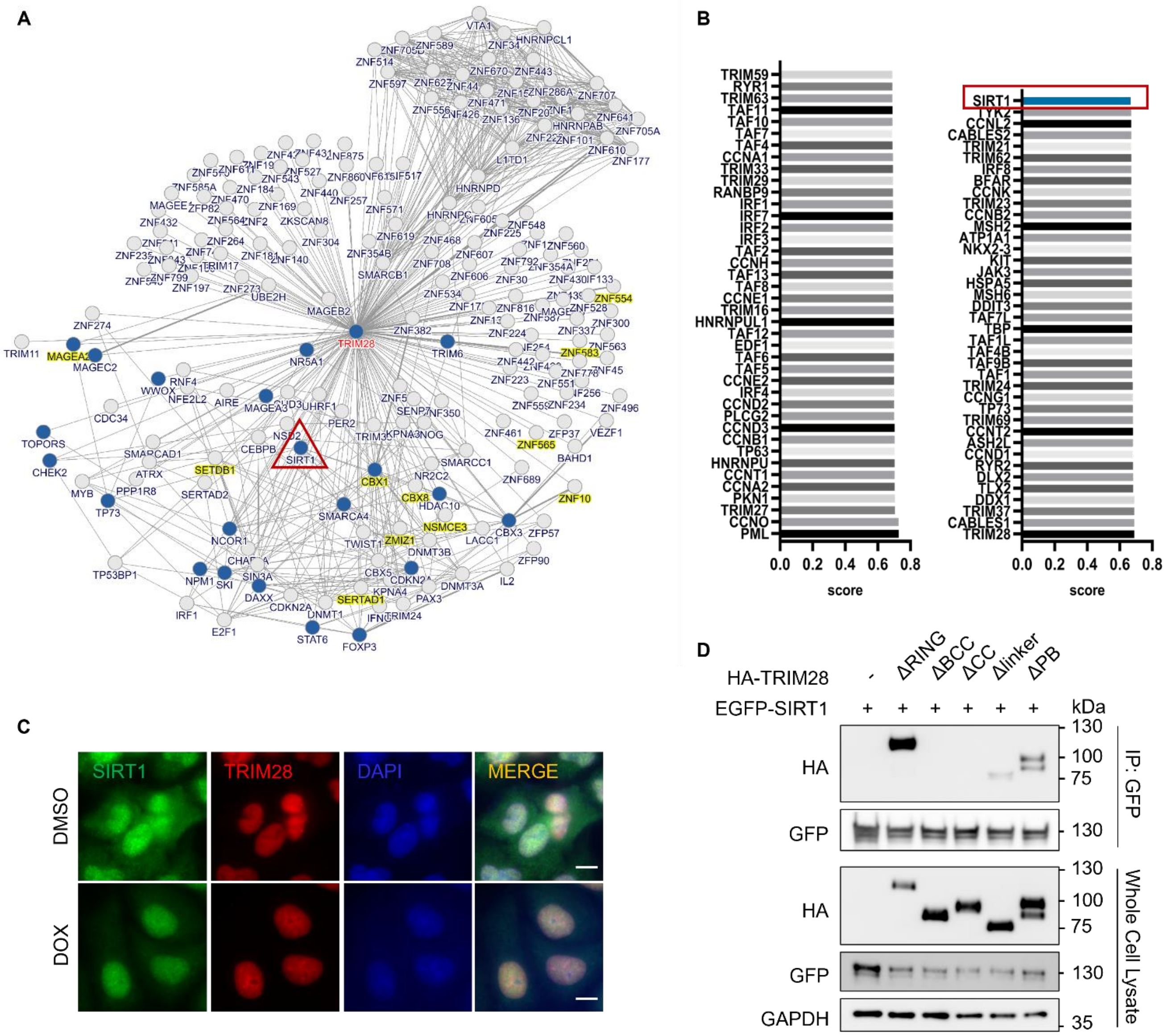
A
TRIM28
SIRT1
B
TRIM59
RYR1
TRIM63
TAF11
TAF10
TAF7
TAF4
CCNA1
TRIM33
TRIM29
RANBP9
IRF1
IRF7
IRF2
IRF3
TAF2
CCNH
TAF13
TAF8
CCNE1
TRIM16
HNRNPUL1
TAF12
EDF1
TAF6
TAF5
CCNE2
IRF4
CCND2
PLCG2
CCND3
CCNB1
TP63
HNRNPU
CCNT1
CCNA2
PKN1
TRIM27
CCNO
PML
0.0 0.2 0.4 0.6 0.8
score
SIRT1
CCNL2
CABLES2
TRIM21
TRIM62
IRF8
BFAR
CCNK
TRIM23
CCNB2
MSH2
ATP1A1
NKX2-3
KIT
JAK3
HSPA5
MSH6
DDIT3
TAF7L
TBP
TAF1L
TAF4B
TAF9B
TAF1
TRIM24
CCNG1
TP73
TRIM69
CCNT2
ASH2L
CCND1
RYR2
DLX2
TLX2
DDX1
TRIM37
CABLES1
TRIM28
0.0 0.2 0.4 0.6 0.8
score
C
SIRT1
TRIM28
DAPI
MERGE
DMSO
DOX
D
HA-TRIM28
-
ΔRING
ΔBCC
ΔCC
Δlinker
ΔPB
EGFP-SIRT1
+ + + + + +
kDa
HA
130
100
75
GFP
130
IP: GFP
HA
130
100
75
GFP
130
GAPDH
35
Whole Cell Lysate

Figure S1 SIRT1 colocalizes and interacts with its potential E3 ubiquitin ligase TRIM28. Related to Figure 3.

(A) Network view of proteins interacted with TRIM28 generated by the inBio Discover software.

(B) Bar chart showing the potential substrates of E3 ubiquitin ligase TRIM28 predicted by the UbiBrower software.

(C) HeLa cells were treated with DOX (5μM) for 8 h, before fixation with 4% PFA and subjected to immunofluorescence analysis with anti-SIRT1 and anti-TRIM28 antibodies, respectively. Scale bar: 10 μm.

(D) HeLa cells were co-transfected with EGFP-SIRT1 and different HA-tagged TRIM28 truncating mutations for 24 h. Cell lysates were subjected to GFP IP and blotted with indicated antibodies.

A

	sgRNAs
Oligo pair	Forward: CACCGGAGCGCTTTTCGCCGCCAG<br>Reverse: AAACCTGGCGGCGAAAAGCGCTCC
	Forward: CACCgCGCTGCGGGATAATGGTCGG<br>Reverse: AAACCCGACCATTATCCCGCAGCGC
	Forward: CACCgCTTCGAGACGCGCATGAACG<br>Reverse: AAACCGTTCATGCGCGTCTCGAAGC

B

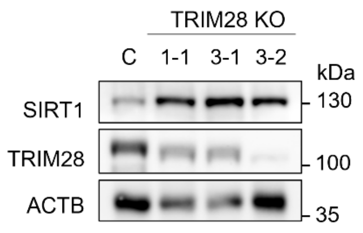

C

D

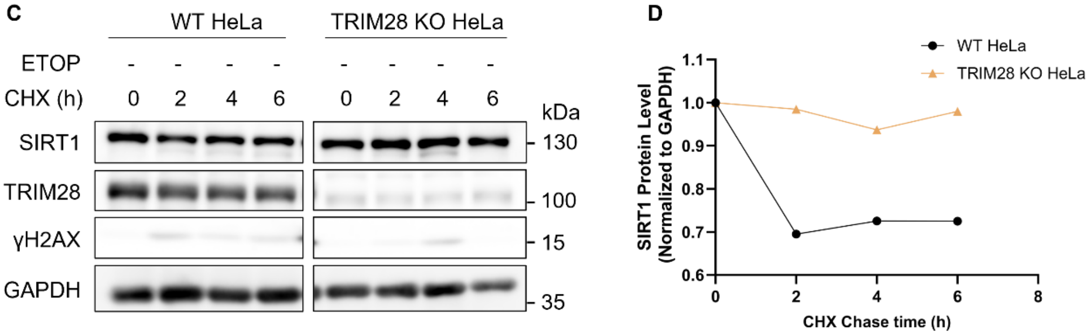

Figure S2 TRIM28 knockout in HeLa cells stabilizes SIRT1. Related to Figure 4.
(A) Three sgRNAs predicted by online tool https://crispr.cos.uni-heidelberg.de/ for knocking out TRIM28 in HeLa cells using CRISPR/Cas9 system.
(B) Selected single clones were collected and subjected to western blot analysis with specific antiSIRT1 and anti-TRIM28 antibodies.
(C) HeLa cells and TRIM28 KO HeLa cells were treated with protein synthesis inhibitor cycloheximide (CHX, 150μg/ml). Cells were collected every two hours and subsequently subjected to western blot analysis to determine the half-life of SIRT1 in this pulse chase experiment.
(D) Quantification of SIRT1 protein degradation rate in (C), as performed by the Fiji software.

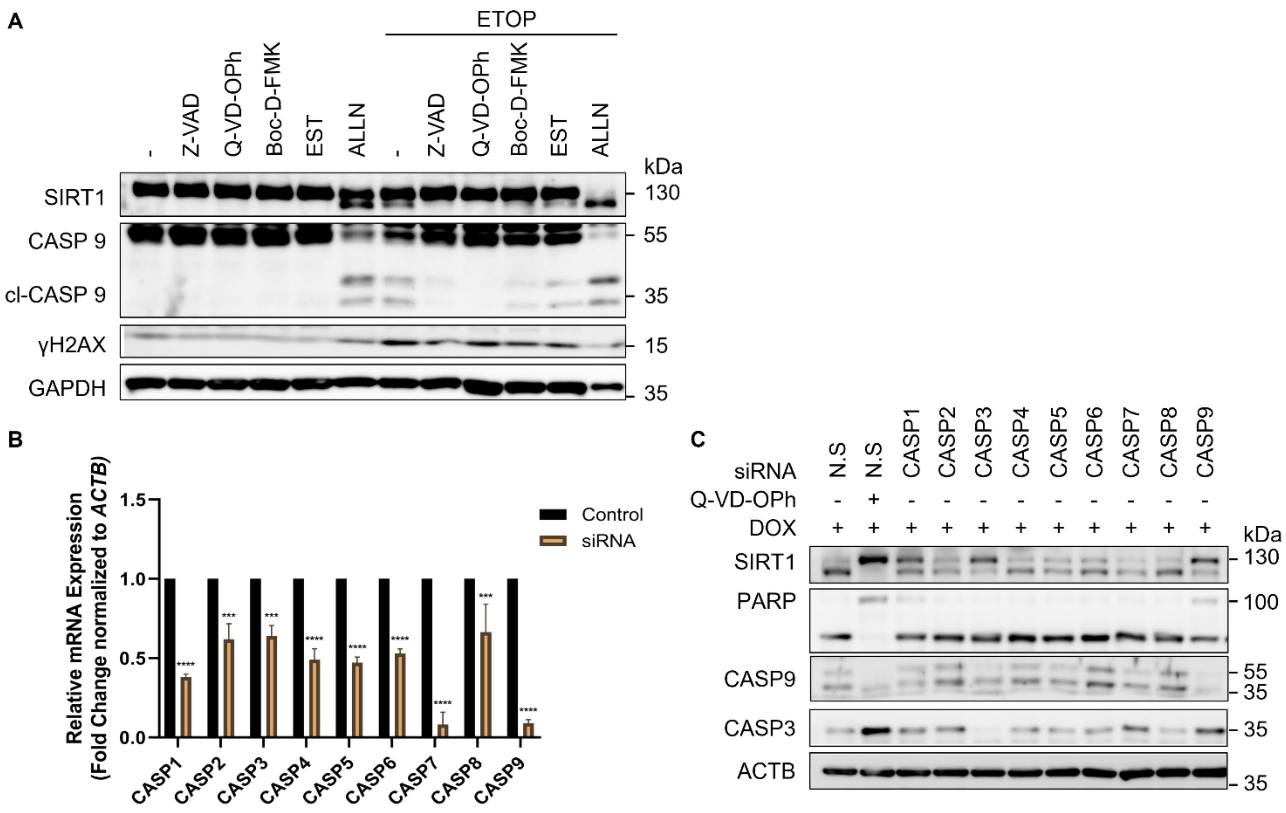

D

Caspase	Position	Site	N fragment	C fragment	Frequency score	Similarity maxscore	Average score	Specificity
1	**704-709**	**DEPD.VP**	**77.5 kD**	**4.2 kD**	**0.714**	**82.857**	**59.185**	**>99.9%**
	431-436	DEVD.LL	47.3kD	34.3 kD	0.714	58.065	41.475	>99.9%
	286-291	DFPD.LP	30.6 kD	51.1 kD	0.595	60.000	35.714	>99.9%
3	431-436	DEVD.LL	47.3 kD	34.3 kD	8.953	79.310	41.475	>99.9%
	**704-709**	**DEPD.VP**	**77.5 kD**	**4.2 kD**	**1.496**	**82.857**	**59.185**	**>99.9%**
	564-569	DDLD.VS	61.9 kD	19.8 kD	0.868	83.333	72.322	>99.9%
6	122-127	DEDD.DD	12.3 kD	69.4 kD	25.558	91.176	2330.31 5	>99.9%
	123-128	EDDD.DE	12.4 kD	69.3 kD	10.564	88.571	935.625	>99.9%
	431-436	DEVD.LL	47.3 kD	34.3 kD	12.810	65.517	839.295	>99.9%
7	431-436	DEVD.LL	47.3 kD	34.3 kD	10.370	67.742	702.509	>99.9%
	163-168	DEED.RA	16.7 kD	64.9 kD	0.212	66.667	14.109	>99.9%
8	431-436	DEVD.LL	47.3 kD	34.3 kD	1.111	66.667	74.074	>99.9%
	**704-709**	**DEPD.VP**	**77.5 kD**	**4.2 kD**	**0.667**	**63.636**	**42.424**	**>99.9%**
	283-288	LAVD.FP	30.2 kD	51.5 kD	0.427	71.875	30.716	>99.9%

Figure S3 Caspases target SIRT1 for cleavage in DDR. Related to Figure 5.

(A) HeLa cells were treated with DMSO or ETOP (150µM) for 16 h with or without z-VAD (20µM), Q-VDOPh (20µM), Boc-D-FMK (20µM), EST (20µM), or ALLN (50µM). Cells were harvested for western blot analysis.

(B) HeLa cells were transfected with siCASP1-9 respectively for 48 hours, total RNA was extracted and the mRNA levels of the caspases were quantified by qRT-PCR assay with specific primers, while normalized by house-keeping gene ACTB. (Columns, mean; error bars, ± S.D; ns, not significant; ***$P<0.0002$ and ****$P<0.0001$; Two-tailed Student`s t-test) (n=3).

(C) HeLa cells were transfected with siCASP1-9 respectively for 48 hours before treated with DOX (5µM) for 16 h. Q-VD-OPh (20µM) treated cells were used as a positive control. Cells were subsequently lysed for western blot analysis.

(D) Table showing SIRT1 cleavage site prediction targeted by caspase 1/3/6/7/8. SitePrediction tool http://www.dmbr.ugent.be/prx/bioit2-public/SitePrediction/ was used.

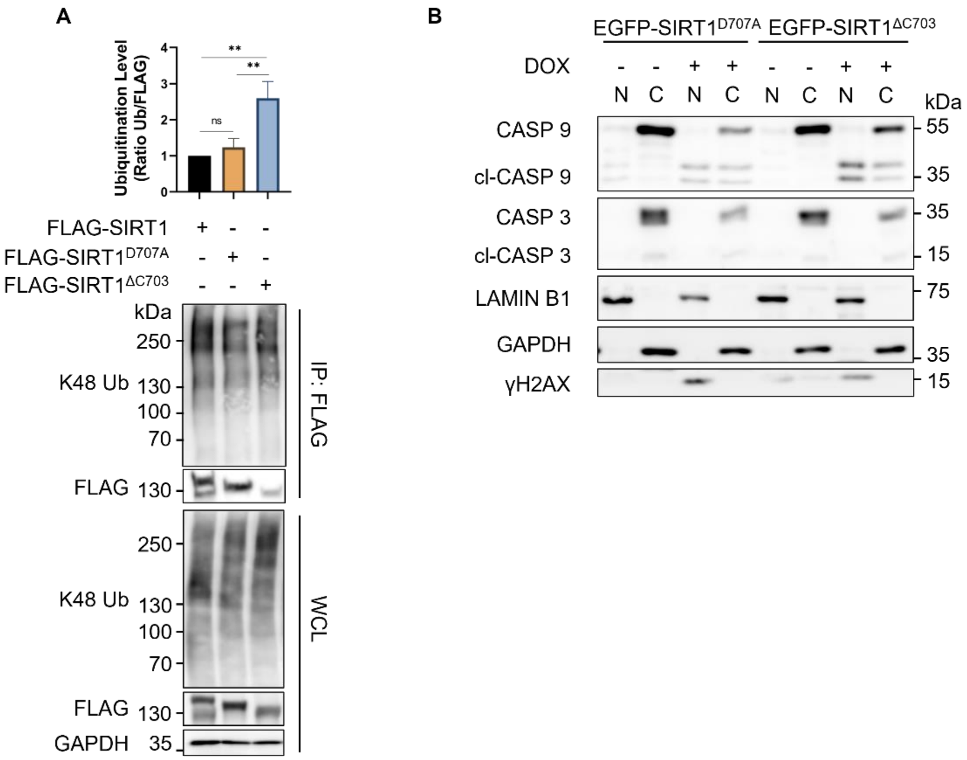

A
Ubiquitination Level (Ratio Ub/FLAG)
0
1
2
3
4
ns
**
**
FLAG-SIRT1 + - -
FLAG-SIRT1D707A - + -
FLAG-SIRT1ΔC703 - - +
kDa
250
K48 Ub
130
100
70
IP: FLAG
FLAG
130
250
K48 Ub
130
100
70
WCL
FLAG
130
GAPDH
35
B
EGFP-SIRT1D707A
EGFP-SIRT1ΔC703
DOX - - + + - - + +
N C N C N C N C
kDa
CASP 9
55
cl-CASP 9
35
CASP 3
35
cl-CASP 3
15
LAMIN B1
75
GAPDH
35
γH2AX
15

Figure S4 Cleaved SIRT1 has higher ubiquitination level compared to full-length SIRT1. Related to Figure 6.

(A) HeLa cells were transfected with FLAG-SIRT1, FLAG-SIRT1^{D707A} or FLAG-SIRT1$^{\Delta C703}$ for 24 h. Cells were treated with MG132 (10μM) for 2 h before being lysed with denature IP lysis buffer and subjected to immunoprecipitation with anti-FLAG beads. (Columns, mean; error bars, ± S.D; *P <0.0332, n.s, not significant; one-way ANOVA with Tukey`s multiple comparisons) (n=3)

(B) HeLa cells were transfected with EGFP-SIRT1^{D707A} or EGFP-SIRT1$^{\Delta C703}$ for 24 h, then treated with DMSO or DOX (5μM) for 6 h. Cells were harvested and subjected to cell fractionation and western blot analysis. Lamin B1 and GAPDH served as nuclear and cytoplasm markers, respectively.

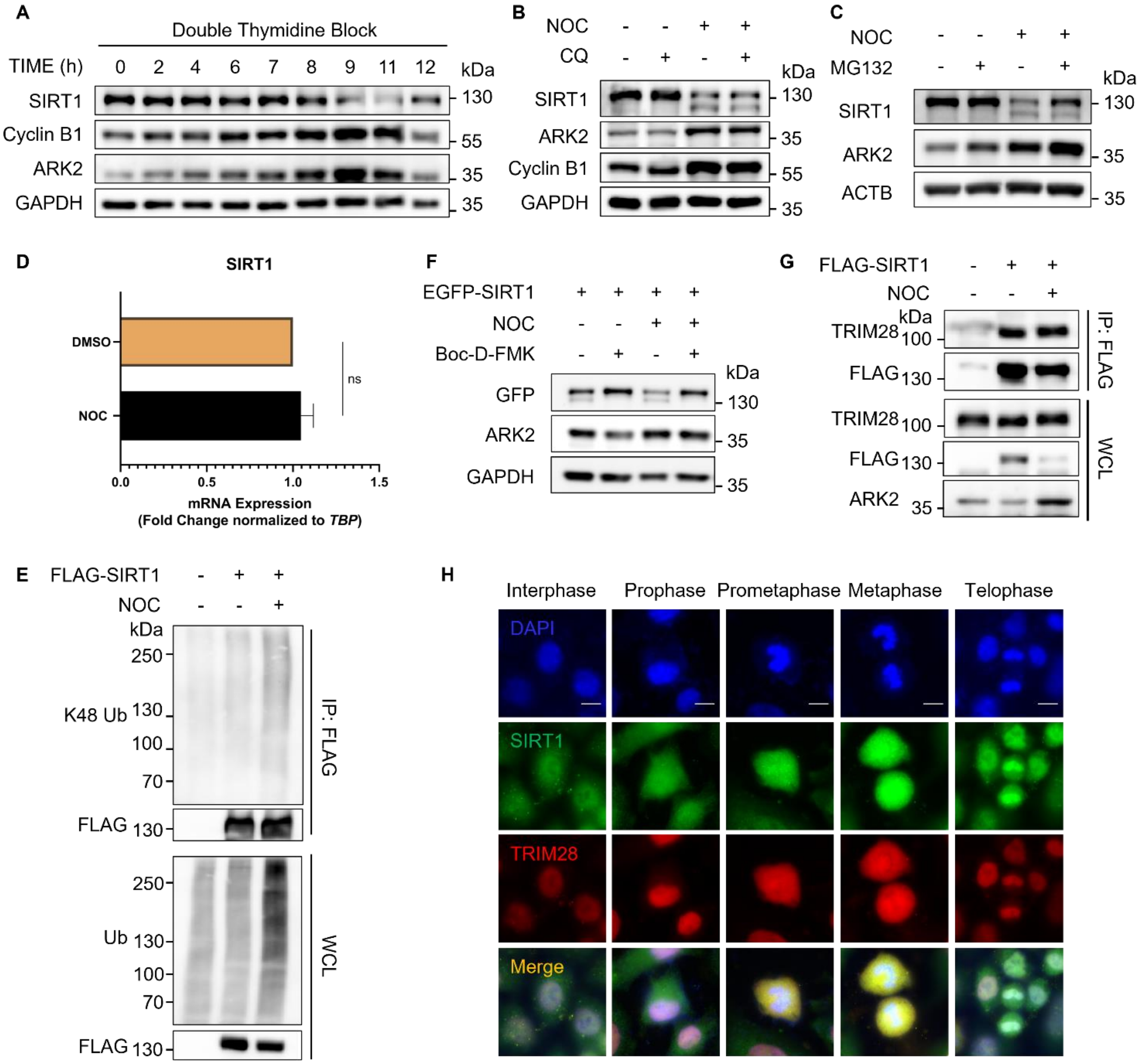
A
Double Thymidine Block
TIME (h) 0 2 4 6 7 8 9 11 12
kDa
SIRT1 130
Cyclin B1 55
ARK2 35
GAPDH 35
B
NOC - - + +
CQ - + - +
kDa
SIRT1 130
ARK2 35
Cyclin B1 55
GAPDH 35
C
NOC - - + +
MG132 - + - +
kDa
SIRT1 130
ARK2 35
ACTB 35
D
SIRT1
DMSO
NOC
ns
0.0 0.5 1.0 1.5
mRNA Expression
(Fold Change normalized to TBP)
F
EGFP-SIRT1 + + + +
NOC - - + +
Boc-D-FMK - + - +
kDa
GFP 130
ARK2 35
GAPDH 35
G
FLAG-SIRT1 - + +
NOC - - +
kDa
TRIM28 100
FLAG 130
IP: FLAG
TRIM28 100
FLAG 130
ARK2 35
WCL
E
FLAG-SIRT1 - + +
NOC - - +
kDa
250
K48 Ub 130
100
70
FLAG 130
IP: FLAG
250
Ub 130
100
70
FLAG 130
WCL
H
Interphase Prophase Prometaphase Metaphase Telophase
DAPI
SIRT1
TRIM28
Merge

Figure S5 SIRT1 degrades during mitosis in a proteasome-mediated way.

(A) Cells were incubated at 37℃ with thymidine for 18 h, washed with PBS and incubated with fresh medium for 9 h before a second round of 18 h thymidine block, then finally released into fresh medium and collected at indicated time points for western-blot analysis.

(B & C) HeLa cells were pretreated with NOC (150ng/ml, 16 h) before addition of CQ (20μM) (B) or MG132 (20μM) (C) for 2 h, then subjected to western blot analysis.

(D) Total RNA was extracted from HeLa cells treated with DMSO or NOC (150ng/ml) for 16 h, and mRNA levels of SIRT1 were quantified by qRT-PCR assay, with normalization by house-keeping gene TBP (TATA-box binding protein). (Columns, mean; error bars, ± S.D; ns, not significant; Two-tailed Student`s t-test) (n=3).

(E) HEK293T cells were transfected with FLAG-SIRT1 for 24 h. Cells were harvested after MG132 (10μM) treatment for 2 h and lysed with denature IP lysis buffer, finally subjected to immunoprecipitation with anti-FLAG agarose.

(F) HeLa cells were transfected with EGFP-SIRT1 for 24 h, treated with NOC (150ng/ml) and Boc-D-FMK (20μM) separately or collaboratively as indicated for 16 h. Cells were harvested and subjected to western blot analysis.

(G) HEK293T cells were transfected with FLAG-tagged SIRT1 for 24 h and then treated with DMSO or NOC (150ng/ml) for 16 h. Cell lysates were subjected to FLAG IP and blotted with indicated antibodies.

(H) HeLa cells fixed with 4% PFA for immunofluorescence analysis with anti-SIRT1 and anti-TRIM28 antibodies. Cells undergoing the cell cycle phases of interphase and mitosis (prophase, prometaphase, metaphase, and telophase included) were observed and imaged under the Leica microscope. Scale bar: 20 μm.

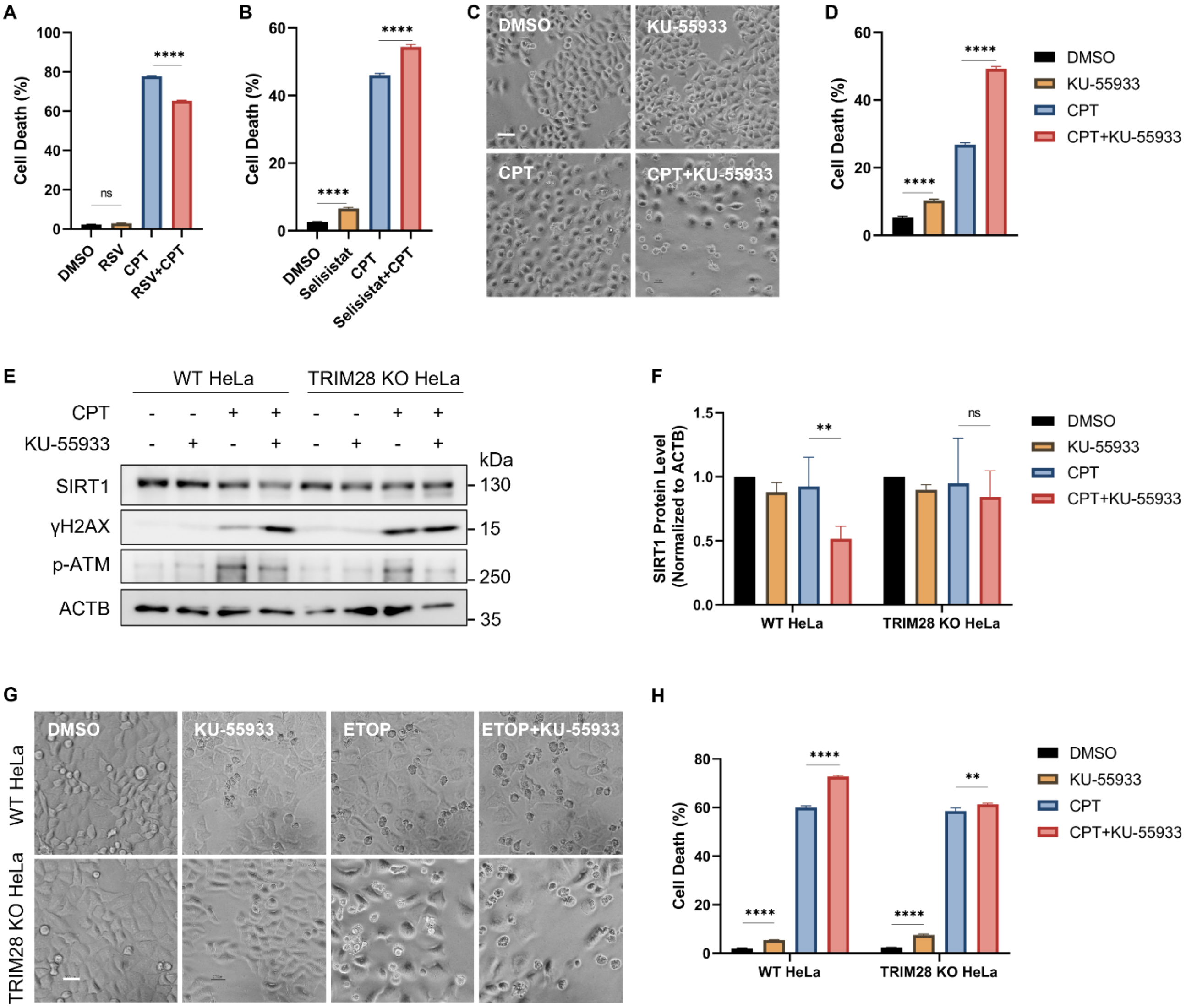
A
Cell Death (%)
100
80
60
40
20
0
ns
****
DMSO
RSV
CPT
RSV+CPT
B
Cell Death (%)
60
40
20
0
****
****
DMSO
Selisistat
CPT
Selisistat+CPT
C
DMSO
KU-55933
CPT
CPT+KU-55933
D
Cell Death (%)
60
40
20
0
****
****
DMSO
KU-55933
CPT
CPT+KU-55933
E
WT HeLa
TRIM28 KO HeLa
CPT
KU-55933
kDa
SIRT1
130
γH2AX
15
p-ATM
250
ACTB
35
F
SIRT1 Protein Level (Normalized to ACTB)
1.5
1.0
0.5
0.0
**
ns
WT HeLa
TRIM28 KO HeLa
DMSO
KU-55933
CPT
CPT+KU-55933
G
DMSO
KU-55933
ETOP
ETOP+KU-55933
WT HeLa
TRIM28 KO HeLa
H
Cell Death (%)
80
60
40
20
0
****
****
****
**
WT HeLa
TRIM28 KO HeLa
DMSO
KU-55933
CPT
CPT+KU-55933

Figure S6 ATM inhibition increases SIRT1 degradation upon DNA damage in a TRIM28-dependent manner. Related to Figure 7.

(A) HeLa cells were treated with resveratrol (RSV, SIRT1 activator, 50µM) and CPT (10µM) separately or collaboratively for 24 h, before cell death analysis using propidium iodide (PI) staining and flow cytometry. Data was analyzed by the FlowJo software.

(B) HeLa cells were treated with Selisistat (SIRT1 inhibitor, 25µM) and CPT (10µM) separately or collaboratively for 24 hours, before cell death analysis using PI staining and flow cytometry. Data was analyzed by the FlowJo software.

(C & D) HeLa cells were treated with either KU-55933 (25µM) alone or together with CPT (5µM) for 24 h.

(C) Morphology of the cells was observed and captured under a phase-contrast microscope. Scale bar: 200 µm.

(D) Cells were then subjected to cell death analysis using PI staining and flow cytometry. Data was analyzed by the FlowJo software.

(E) WT HeLa cells and TRIM28 KO HeLa cells were treated with either KU-55933 (25µM) alone or together with CPT (5µM) for 6 h, before western blot analysis.

(F) Quantification of SIRT1 levels in (E) performed by the Fiji software. Error bars indicate the mean ± S.D. Representative results of four biological independent replicates are shown (n=4).

(G) HeLa cells and TRIM28 KO HeLa cells were treated with KU-55933 (25µM) or ETOP (150µM) separately or collaboratively for 24 h. Morphology of the cells was observed and captured under phase contrast microscopy. Scale bar = 200 µm.

(H) HeLa cells and TRIM28 KO HeLa cells were treated with KU-55933 (25µM) or CPT (5µM) separately or collaboratively for 24 h, before cell death analysis using PI staining and flow cytometry.

One-way ANOVA with Tukey`s multiple comparisons; Mean ± S.D.; ns, not significant; *P<0.0332, **P<0.0021, ***P<0.0002, and ****P<0.0001; Representative results of four biological replicates are shown (n=5).

Supplementary Table 1 Primers for qRT-PCR (Related to Methods)

Name	Sequence (5`-3`)
**Human *SIRT1***	Forward: TAGCCTTGTCAGATAAGGAAGGA<br>Reverse: ACAGCTTCACAGTCAACTTTGT
**Human *ACTIN***	Forward: CATGTACGTTGCTATCCAGGC<br>Reverse: CTCCTTAATGTCACGCACGAT
**Human *TBP***	Forward: CCACTCACAGACTCTCACAAC<br>Reverse: CTGCGGTACAATCCCAGAACT
**Human *TRIM28***	Forward: TTTCATGCGTGATAGTGGCAG<br>Reverse: GCCTCTACACAGGTCTCACAC
**Human *CASP 1***	Forward: GGAAACAAAAGTCGGCAGAG<br>Reverse: ACGCTGTACCCCAGATTTTG
**Human *CASP 2***	Forward: GGTGATGGTCCTCCCTGTCT<br>Reverse: TACTCATCACCAGTGCCAAGC
**Human *CASP 3***	Forward: ATGGGAGCAAGTCAGTGGAC<br>Reverse: CGTACCAGAGCGAGATGACA
**Human *CASP 4***	Forward: AAGAGAAGCAACGTATGGCAGGAC<br>Reverse: GGACAAAGCTTGAGGGCATCTGTA
**Human *CASP 5***	Forward: GGTGAAAAACATGGGGAACTC<br>Reverse: TGAAGAACAGAAAGCAATGAAGT
**Human *CASP 6***	Forward: CCAGACAGACAAGCTGGACA<br>Reverse: TGTACCAGGAGCCATTCACA
**Human *CASP 7***	Forward: CCGAGTGCCCACTTATCTGT<br>Reverse: ACCTGTCGCTTTGTCGAAGT
**Human *CASP 8***	Forward: TTCTCCCTACAGGGTCATGC<br>Reverse: GCAGGCTCAAGTCATCTTCC
**Human *CASP 9***	Forward: TTCCCAGGTTTTGTCTCCTG<br>Reverse: GGGACTGCAGGTCTTCAGAG
